# A role for telomere length and chromosomal damage in idiopathic pulmonary fibrosis

**DOI:** 10.1186/s12931-018-0838-4

**Published:** 2018-07-09

**Authors:** John E. McDonough, Dries S. Martens, Naoya Tanabe, Farida Ahangari, Stijn E. Verleden, Karen Maes, Geert M. Verleden, Naftali Kaminski, James C. Hogg, Tim S. Nawrot, Wim A. Wuyts, Bart M. Vanaudenaerde

**Affiliations:** 1 Laboratory of Respiratory Diseases, Department of Chronic Diseases, Metabolism, and Ageing, KU Leuven, Herestraat 49, O&N I, box 706, B-3000 Leuven, Belgium; 2 Centre for Environmental Sciences, Hasselt University, Hasselt, Belgium; 3 University of British Columbia, Centre for Heart Lung Innovation, Vancouver, BC Canada; 4 Section of Pulmonary, Critical Care, and Sleep Medicine, Yale University, New Haven, CT USA

**Keywords:** IPF, Telomere length, Gamma-H2AX, Fibrosis, Collagen 1, Elastin, microCT

## Abstract

**Background:**

Idiopathic pulmonary fibrosis is a fatal lung disease characterized by a progressive formation of fibroblastic foci in the interstitium. This disease is strongly associated with telomere dysfunction but the extent of telomere shortening and consequent chromosomal damage within IPF lungs and with regional disease severity remains unknown.

**Methods:**

Explanted IPF lungs (*n* = 10) were collected from transplant surgeries with six samples per lung analysed to capture the regional heterogeneity ranging from mild to severe disease. Non-used donor lungs (*n* = 6) were collected as “healthy” controls. Structural changes related to disease severity (microCT surface density), relative telomere length (real-time qPCR), and quantitative histology of chromosomal damage (γ-H2A.X) and extracellular matrix (elastin, total collagen, collagen 1, and collagen 3) were measured. A multivariate linear mixed-effects model controlling for subject was used to identify association of disease severity or fibrotic markers with telomere length and chromosomal damage.

**Results:**

We observed shorter telomere length (*p* = 0.001) and increased chromosomal damage (*p* = 0.018) in IPF lungs compared to controls. In IPF lungs, telomere length was associated with total collagen (*p* < 0.001) but not with structural changes of disease severity. Chromosomal damage was positively associated with increased elastin (*p* = 0.006) and negatively with structural disease severity (*p* = 0.046). Extensive γ-H2A.X staining was also present in airway epithelial cells.

**Conclusions:**

Telomere length and chromosomal damage are involved in IPF with regional variation in telomere length and chromosomal damage associated with pathological changes in tissue structure and the extracellular matrix.

## Background

Idiopathic pulmonary fibrosis (IPF) is a progressive lung disease with a median survival of 2 to 3 years after diagnosis and characterized by a restrictive respiratory function failure caused by formation of fibrotic foci within the lung interstitium [1]. While the etiology of the disease remains unknown, studies have indicated that IPF is strongly associated with a reduction in telomere length. Telomeres are nucleoprotein structures containing thousands of nucleotide tandem-repeats, that stabilize chromosome ends preventing DNA degradation. Following cell division, these repeats are progressively lost resulting in a shortening of the telomeres in older subjects. Consequently, telomeres below a critical threshold result in chromosomal instability and activation of the DNA damage response pathways resulting in cellular senescence and apoptosis [2].

In IPF, mutations in genes related to telomerase (telomere elongating enzyme), including telomerase reverse transcriptase (TERT), regulator of telomere elongation helicase 1 (RTEL1), or poly(A)-specific ribonuclease (PARN), have been found to occur in 8–15% of familial IPF and up to 11.3% of sporadic IPF patients within a lung transplant cohort [3, 4]. Even IPF subjects without known telomerase mutations are found to have shortened telomeres compared to controls, [5] which may be due to rare and uncharacterized variants. Several groups have demonstrated shortened telomeres in the peripheral blood in IPF [6–8] and its association with decreased survival [9, 10]. However, the association between these observations and what is occurring in the lungs is not known. Despite the significance of telomeres in IPF, few studies has directly measured telomere length in the lungs [6, 11]. These studies examined telomere length specifically in type II alveolar cells (AT2) in human IPF lung tissue. The most recent of these studies found sporadic IPF subjects had shortened telomere lengths in AT2 cells in fibrotic regions compared to surrounding cells [11].

Shortened telomeres result in exposure of the chromosomal ends which are then recognized as DNA double strand breaks (DSB) and activate the DNA damage repair (DDR) response [12]. One of the earliest events in DDR is phosphorylation of histone 2AX (H2AX) to form γ-H2A.X, making this a useful marker of DNA damage [13]. While senescence pathways have been associated with IPF, decreased telomere length that results in destabilized chromosomes and leads to an increased number of DNA strand breaks has not been characterized in the human lung.

Due to the convergence between telomere dysfunction, chromosomal DNA damage, and senescence, we sought to study these factors in IPF. The aim of the current study was to correlate telomere length and chromosomal damage with structural changes related to disease severity and the extracellular matrix in human lung tissue with end-stage IPF.

## Methods

### Patient data and sampling

Patients diagnosed with IPF by multidisciplinary consensus who underwent lung transplantation for their disease at UZ Hospital in Leuven, Belgium were selected for this study (*n* = 10). Donor lungs not suitable for transplantation were used as “healthy” controls (*n* = 6). All lungs were collected following local hospital ethical committee approval (ML6385) and informed patient consent. See Table 1 for patient demographics and available lung function information.

**Table 1:** Demographic data for control and IPF subjects

	Control	IPF
N	6	10
Sex (M:F)	6 M: 0 F	10 M: 0 F
Age (year)	57.8 ± 10.7	57.0 ± 5.1
Height (cm)	175.8 ± 5.9	172.6 ± 6.7
Weight (kg)	83.2 ± 13.6	72.9 ± 10.3
BMI	26.8 ± 3.5	24.4 ± 2.2
Smoking History (NS:FS:CS)	4:1:1	0:10:0
Pack Year	FS = 24 CS = 39	22.8 ± 11.8
FEV_1_pp (%)	–	60.7 ± 15.5
FVCpp (%)	–	58.7 ± 19.7
TLCpp (%)	–	54.9 ± 16.9
RVpp (%)	–	50.4 ± 19.9
DL_CO_pp (%)	–	27.6 ± 7.8

*NS* non-smoker, *FS* former smoker, *CS* current smoker, *pp.* % predicted

Explanted lungs were inflated to 30 cm H_2_O then held at 10 cm H_2_O pressure while frozen over liquid nitrogen vapour according to previously established protocols [14]. Lungs were then imaged using a high-resolution CT scanner (Siemens SOMATOM, Germany) and subsequently cut into 2 cm thick slices along the transverse plane with each slice systematically sampled using a 1.4 cm diameter coring drill. From each upper, mid, and lower lung region two cores were randomly selected for a total of 6 samples per lung and 96 samples in total. We identified one sample as mislabelled with the remaining 95 samples used for downstream analysis.

### MicroCT scanning

MicroCT scans on frozen samples allowed for detailed, non-destructive measurements of tissue structure to provide an accurate assessment of pathological structure changes related to disease severity within the tissue. Frozen lung samples were microCT scanned using a Bruker Skyscan 1172 (Bruker, Belgium) with cooling stage. Scans settings were set at 40 kV, 240 mA, and 0.5° rotation step at − 30 °C. Temperature was maintained throughout the scan by placing the sample in a Styrofoam cylinder with dry-ice on top. Scans were reconstructed using NRecon software (Bruker, Belgium) and images analysed using CTAn software (Bruker, Belgium). In the lung, parenchymal structures that enable gas exchange have a high surface to volume ratio (surface density). As collapse of parenchymal structures and development of fibrosis are thought to occur in the pathological progression of IPF, measurements of surface density can be used to approximate the extent of disease within the sample. The image analysis software CTAn was used to measure surface density following manual thresholding to segment tissue from air.

### Histology and immunostaining

Following microCT, a portion of each core was vacuum embedded in O.C.T. (Sakura) [15]. Cryosections were cut from the O.C.T. embedded core for histology at 8 μm thickness onto coated glass slides then dried and fixed with acetone. Histological stainings were performed to examine tissue structure and microscopic honeycombing (H&E), total collagen (picrosirius red), and elastin (Van Gieson).

Immunostaining was performed using anti-gamma H2A.X (phosphor S139) antibody (ab11174) (Abcam, Cambridge, USA) diluted 1/40,000 by an automated stainer (Leica Bond RX, Germany). For extracellular matrix markers, mouse monoclonal anti-Collagen 1 (ab6308) was used at 1/200,000 dilution and rabbit polyclonal anti-Collagen 3 (ahp1848) (Bio-Rad, USA) at 1/100 dilution.

Slides were imaged by an Aperio digital pathology slide scanner (Leica Biosystems Inc., Canada) and quantified using the Aperio Image Analysis software suite (Leica Biosystems Inc., Canada). A threshold was applied to each image to separate tissue from air while a separate threshold was set to identify positively stained tissue from surrounding tissues. A volume fraction (Vv) of staining for each slide was calculated as the fraction of stained tissue area per total tissue area.

We performed pathological assessment on H&E slides to exclude samples with emphysema, comprised predominantly of large vessels or airways, or artefacts from processing that may confound analysis. A total of 22 samples were excluded with the remaining 73 samples used for statistical analysis (44 IPF and 29 control samples).

### Relative telomere length measurement

Tissue sections from O.C.T. embedded samples were used for DNA extraction using the QIAamp DNA Mini Kit (Qiagen, Inc., Venlo, Netherlands). DNA quantity and purity were assessed by Nanodrop 1000 spectrophotometer (Isogen, Life Science, Belgium). DNA integrity was assessed by agarose gel-electrophoresis. Average relative telomere length was measured by a modified quantitative real-time PCR protocol as described previously [16, 17]. Telomere reaction mixture contained 1× QuantiTect SYBR Green PCR master mix (Qiagen, Inc., Venlo, Netherlands), 2 mM dithiothreitol (DTT), 300 nM telg primer (ACACTAAGGTTTGGGTTTGGGTTTGGGTTTGGGTTAGTGT) and 900 nM telc primer (TGTTAGGTATCCCTATCCCTATCCCTATCCCTATCCCTAACA). Cycling conditions were: 1 cycle at 95 °C for 10 min, followed by 2 cycles at 94 °C for 15 s and 49 °C for 2 min and 30 cycles at 94 °C for 15 s, 62 °C for 20 s, and 74 °C for 1 min and 20 s. The single-copy gene (36B4) reaction mixture contained 1× QuantiTect SYBR Green PCR master mix, 300 nM 36B4u primer (CAGCAAGTGGGAAGGTGTAATCC) and 500 nM 36B4d primer (CCCATTCTATCATCAACGGGTACAA). Cycling conditions were: 1 cycle at 95 °C for 10 min, followed by 40 cycles at 95 °C for 15 s, and 58 °C for 1 min and 20 s. Measurements were all performed in triplicate on a 7900HT Fast Real-Time PCR System (Applied Biosystems) in a 384-well format. To assess PCR efficiency a 6-point serial dilution was included on each run. PCR efficiency of the telomere run was 108% and the efficiency for the single-copy gene run was 99%. Relative average telomere lengths (RTL) were expressed as the ratio of telomere copy number to single-copy gene number (T/S) relative to the average T/S ratio of the entire sample set. Coefficients of variation (CV) within triplicates of the telomere run, single-copy gene run, and T/S ratios were 0.6, 0.5, and 7.5% respectively.

### Statistical analysis

As the heterogeneous distribution of disease in IPF would preclude a single sample to be representative of the lung, the mean value for measurements from all samples of each subject was calculated to compare IPF subjects with controls. Data were log2 (RTL) or square root transformed (γ-H2A.X) following Shapiro-Wilk normality testing to ensure a normal distribution for linear analysis. Multivariate linear mixed-effect models were used to compare surface density or fibrotic markers with γ-H2A.X, RTL, and age in the IPF group. Subject was set as a random effect due to using multiple samples per subject and age as an interaction term with RTL due to the association of telomere length with age. All models included acute exacerbations as a confounding variable while DNA yield, a surrogate measure of cellular content within the sample, was included in multivariate models comparing telomere length or chromosomal damage with ECM measurements. Associations identified as significant were analysed using univariate linear regression to determine correlation coefficient. Analyses were conducted using R software (version 3.3.2) with data presented as mean ± standard deviation and graphs displayed as boxplots with median and quartiles or scatterplots showing linear regression line and 95% confidence intervals.

## Results

IPF and control subjects were males of similar age, height, and weight. (Table 1) All IPF subjects were former smokers who had quit smoking from 1 to 30 years with number of packs of cigarettes smoked ranging from 4 to 38 pack years. In the control group, 2 of the subjects were smokers with 1 former smoker at 24 pack years and 1 current smoker at 39 pack years. Despite the extensive smoking history of these two control subjects, RTL and γ-H2A.X levels remained the same as the non-smoker controls.

**Fig. 1:**
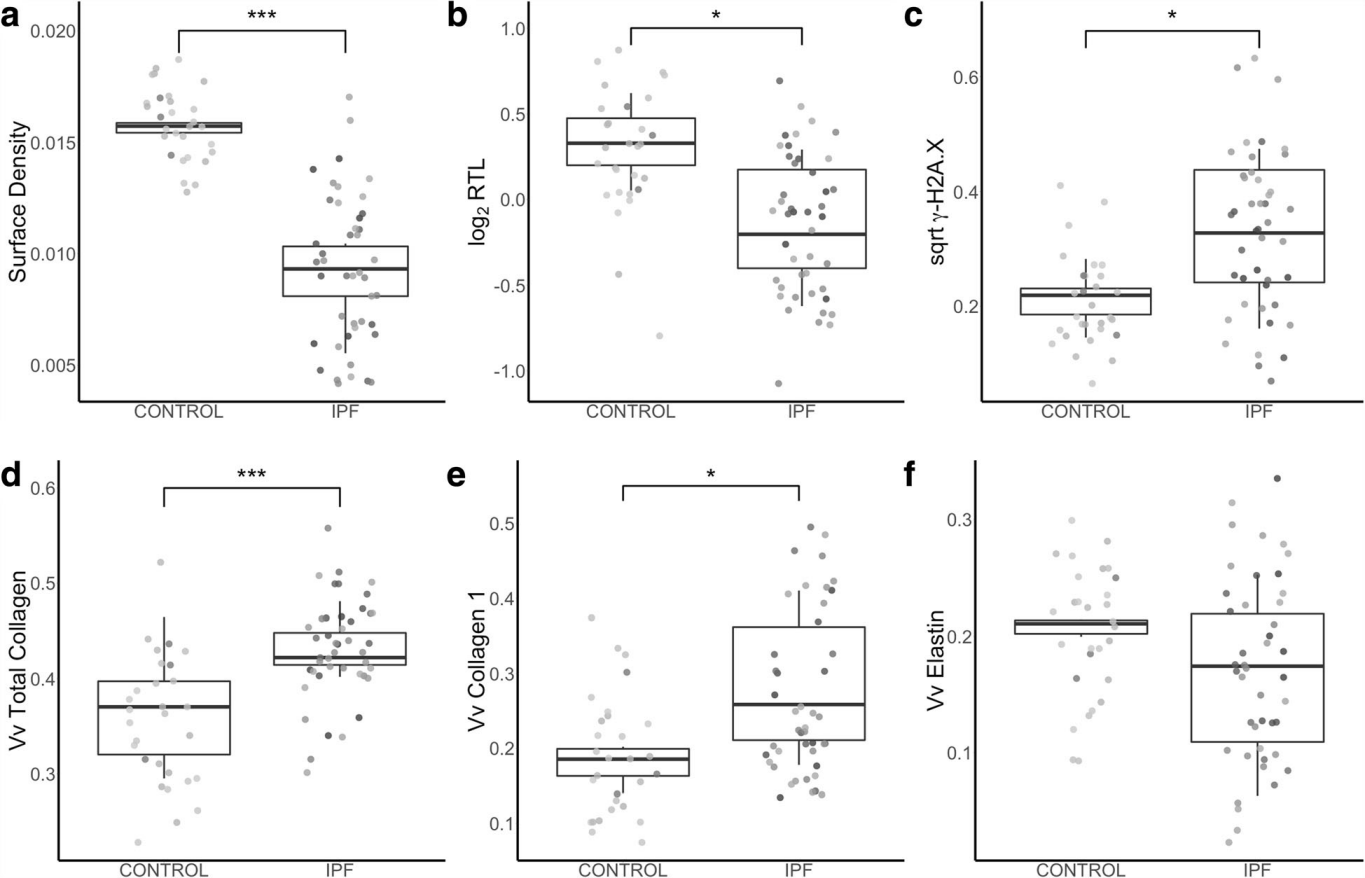
Comparison between control and IPF subjects for (**a**) surface density, (**b**) relative telomere length (RTL) and volume fractions of (**c**) γ-H2A.X, (**d**) total collagen, (**e**) collagen 1, and (**f**) elastin. Grey scale colour identifies each individual subject. Points represent measurements for each sample while boxplot shows distribution of the mean measurement from each subject. * *p* < 0.5, *** *p* < 0.001

Loss of normal parenchymal structures in IPF was seen as decreased surface density in IPF compared to control (control: 0.0157 ± 0.0007 μm, IPF: 0.0092 ± 0.0022 μm; *p* < 0.001). (Figure 1a) PCR based measurement of telomere length demonstrated a ~ 27% reduction in relative telomere length (RTL) in IPF lungs compared to controls (control: 1.27 ± 0.19, IPF: 0.92 ± 0.21; *p* = 0.001). (Figure 1b) Immunostaining of γ-H2A.X showed a 3-fold increase in tissue fraction stained in IPF compared to controls (control: 0.047 ± 0.020, IPF: 0.121 ± 0.079; *p* = 0.018). (Figure 1c) Increased markers of fibrosis were present in IPF compared to control lungs as would be expected. Total collagen (control: 0.37 ± 0.06, IPF: 0.43 ± 0.03; *p* < 0.001) (Fig. 1d) and collagen 1 (control: 0.19 ± 0.04, IPF: 0.28 ± 0.09; *p* = 0.014) (Fig. 1e) were both increased in IPF while no change was noted in elastin (control: 0.21 ± 0.03, IPF: 0.16 ± 0.07; *p* = 0.14) (Fig. 1f) or collagen 3 (control: 0.24 ± 0.05, IPF: 0.23 ± 0.02; *p* = 0.59) (not shown).

**Fig. 2:**
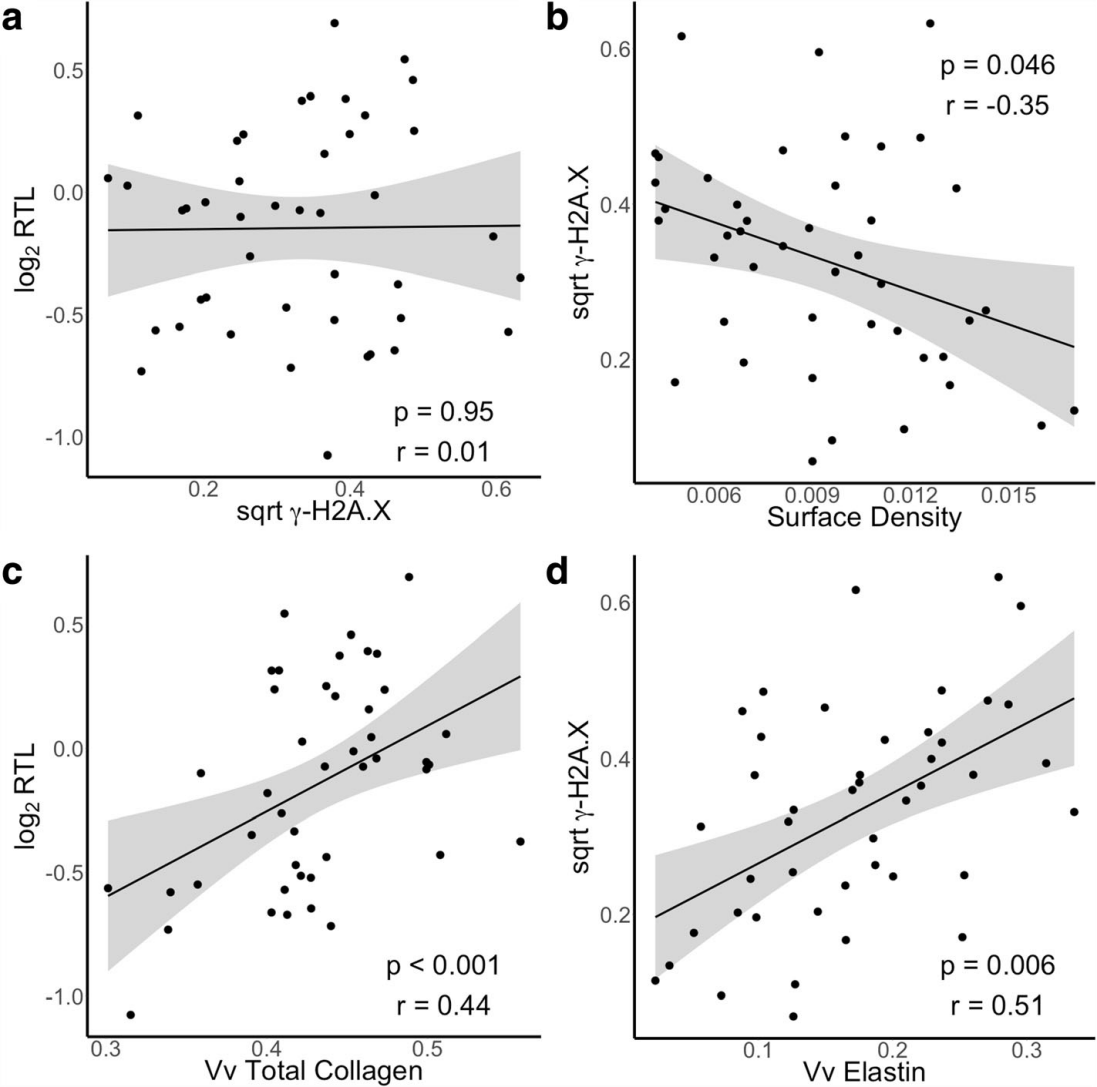
Scatter plots and regression lines for RTL and γ-H2A.X measurements in samples from IPF subjects. **a** No correlation was found between RTL and γ-H2A.X (*p* = 0.95, *r* = 0.01). **b** Negative correlations were found for surface density and γ-H2A.X (*p* = 0.046, *r* = − 0.35). Positive correlations were found for (**c**) relative telomere length and volume fraction of total collagen (p < 0.001, *r* = 0.44) and (**d**) γ-H2A.X and volume fraction elastin (*p* = 0.006, *r* = 0.51). *P*-values were calculated from multivariate linear mixed-effects model. R correlation coefficients were based on univariate linear regression

**Fig. 3:**
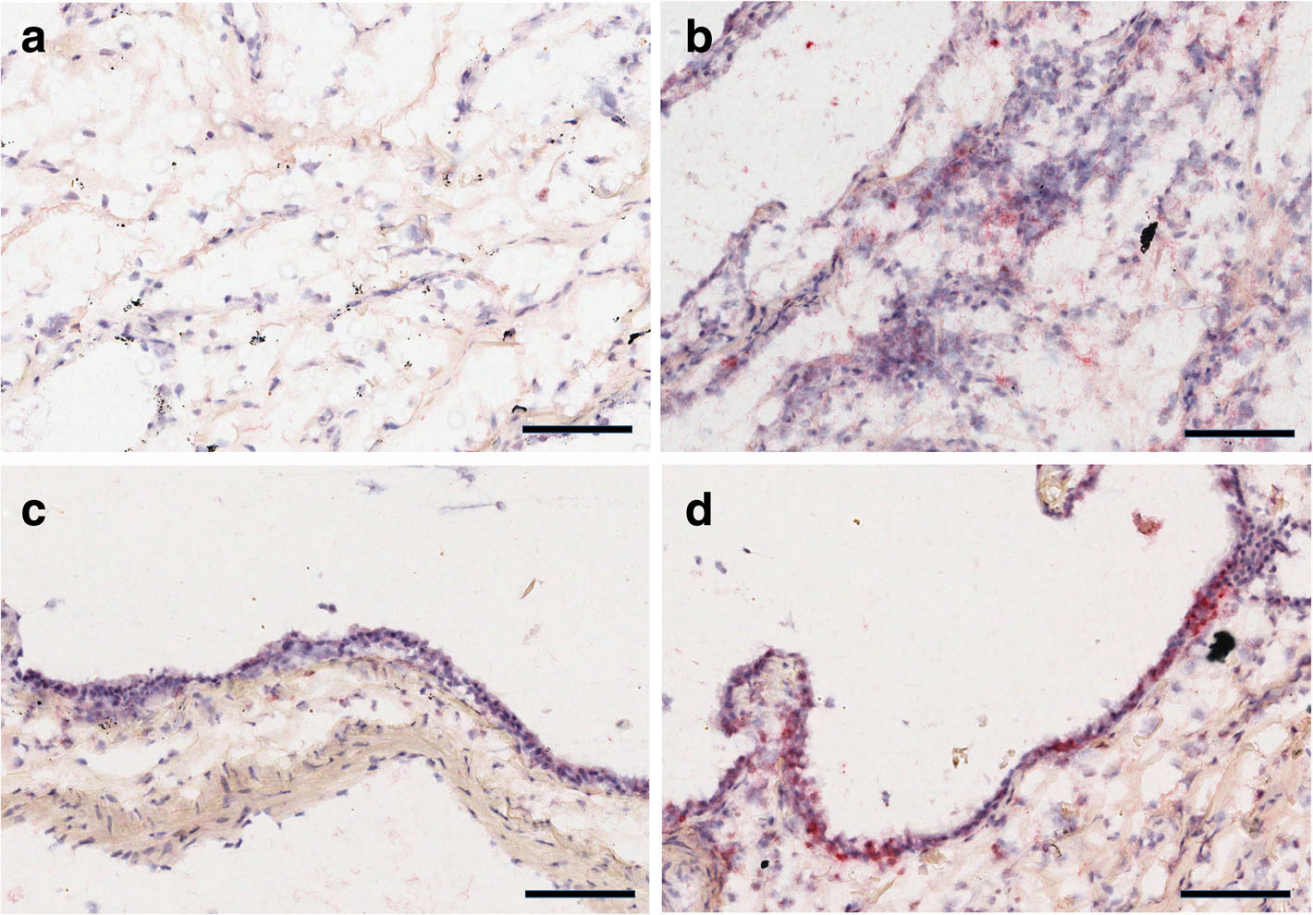
Staining of γ-H2A.X in regions with low surface density and high (**a**) collagen 1 or (**b**) elastin staining. More extensive γ-H2A.X staining (red) was present in the regions with high elastin content. The bronchial epithelium had extensive γ-H2A.X positive staining in the IPF group, primarily in the airway epithelial cells. **c** Airway wall from a control subject. **d** Airway wall from a subject with IPF. Scale Bar = 100 μm

Despite chromosomal damage being one of the consequences of telomere shortening, we found no correlation between regional measurements of RTL with γ-H2A.X in the IPF group (*p* = 0.95, *r* = 0.01). (Figure 2a) Multivariate linear mixed-effects models correlated surface density with γ-H2A.X (*p* = 0.046, *r* = − 0.35) (Fig. 2b) but not age-adjusted RTL (*p* = 0.40, *r* = − 0.06). In a univariate analysis, we found a strong correlation between DNA yield and surface density (R − 0.62; *p* = 5.0E-6) reflecting the increased amounts of tissue/cells within these samples and validating use of DNA yield as a measure of cellular content. Including DNA yield in our multivariate models in measuring extracellular matrix components, we found total collagen was associated with RTL (*p* < 0.001, *r* = 0.44) (Fig. 2d) and γ-H2A.X was associated with both collagen 1 (*p* = 0.003, *r* = − 0.51) (Fig. 2e) and elastin (*p* = 0.006, *r* = 0.51). Elastin and collagen 1 showed an inverse correlation (*p* = 0.01, *r* = − 0.55) (Fig. 2c) and as both were correlated with γ-H2A.X, we applied a multivariate model incorporating both matrix markers with subject as a random effect. In this model, we found γ-H2A.X was significantly correlated with elastin (*p* = 0.035) but not collagen 1 (*p* = 0.76). (Figure 3a-b) No correlations were found for RTL or γ-H2A.X with collagen 3. We also found no correlation between telomere length or γ-H2A.X with regional variation in surface density or matrix proteins in the control samples.

We further examined tissue sections for microscopic honeycombing but found no relationship with either RTL (*p* = 0.51) or γ-H2A.X (*p* = 0.11). Nor were any correlations found between pack years of smoking and RTL (*p* = 0.81) or γ-H2A.X (*p* = 0.18) in the IPF group. Within our IPF cohort, 4 of 10 patients experienced an acute exacerbation prior to transplantation. Including this factor within our model found that exacerbations had no effect on the associations between telomere length or chromosomal damage with ECM or surface density within our samples. In examining the tissue sections, we noted extensive γ-H2A.X staining was present in the bronchial epithelium (Fig. 3c-d).

## Discussion

In this study, we identified shorter telomere lengths and increased chromosomal damage in the lungs of patients with IPF and compared these measurements to regional disease severity in the IPF lungs based on pathologic structural changes associated with parenchymal collapse and fibrosis. Telomere length was not associated with surface density but was positively associated with fibrosis with longer telomere lengths present in regions with increased total collagen. DNA damage was negatively associated with structural changes (surface density) in the lung and positively correlated with elastin but not with collagens reflecting an association with parenchymal collapse rather than fibrosis.

Current theories of the development of IPF suggest the disease initiates in regions of parenchymal collapse which is followed by the formation of fibrotic foci. Decreased surface density as measured by microCT can identify loss of the normal parenchymal structures that characterize both parenchymal collapse or fibrosis. As elastin is a primary component of the alveolar parenchyma and fibrotic lesions are mainly comprised of collagens, staining of these extracellular matrix markers would allow us to differentiate between these two processes. Therefore, parenchymal collapse would be characterized by low surface density and increased elastin content while fibrotic regions would be characterized by low surface density and increased collagen content. We applied this model to our findings and suggest a novel sequence of events involving shortened telomeres and chromosomal damage in disease progression.

Animal models have also found shortened telomeres alone do not result in increased chromosomal damage or fibrosis but mice with a genetic telomere dysfunction displayed increased sensitivity to bleomycin-induced chromosomal damage with subsequent fibrosis [18]. This finding showed that shortened telomeres are an intrinsic and likely systemic dysfunction that provides an environment where extrinsic factors can readily induce chromosomal DNA damage. In our data, we found both decreased telomere length and increased chromosomal damage in IPF subjects but found no relationship between these two variables, supporting the notion that telomere length and chromosome damage may be responding to independent factors.

While smoking is one risk factor in the development of IPF and can induce DNA damage, we found no association between pack years of smoking and the extent of chromosomal damage. However, this was limited by the small sample size and high variability in smoking history of our subjects. A recent study found bacteria may contribute to persistent injury in IPF [19]. while another study has found an association with air pollution and early fibrotic lesions [20]. Though whether these insults contribute to chromosomal damage remains unknown. We propose the hypothesis that chromosomal damage is not due to a single causative agent but to cumulative damage over a lifetime of exposure to multiple factors.

We found increased γ-H2A.X was correlated with increased elastin staining and to structural changes in the IPF lung as measured by decreased microCT surface density but not collagens. As parenchymal collapse before initiation of fibrosis is suggested to be an early events in the development of IPF [21] these data suggest chromosomal damage is related to the parenchymal collapse in the early stage of IPF and not with the fibrosis present at later stages of the disease, similar to what has been found in animal models [18].

Increased fibrosis, measured by total collagen, was correlated with increased telomere length, this paradoxical finding can be explained by studies that have shown type II alveolar epithelial (AT2) cells are decreased in the fibroblastic foci [22] and these cells have significantly shorter telomeres than neighbouring cells [6, 11]. Together, loss of the AT2 cells in the fibroblastic regions would increase the average telomere length of remaining cells (i.e. fibroblasts), in line with our findings. However, honeycombing, usually present in end-stage fibrosis, was not correlated with either RTL or γ-H2A.X in our samples.

One limitation of this study was the use of explant lungs to measure telomere length. While use of these lungs was necessary to document the regional differences in telomere length and chromosomal damage, the patients included in this study have a more rapidly progressive disease and would be biased towards those with telomere dysfunction [23]. Therefore, our findings may overestimate the effects of telomere length in the general IPF patient population but is likely representative of the more severe phenotype of IPF. We also used a real-time PCR method to determine telomere lengths, which has higher assay variability compared to the traditionally used Southern blot terminal restriction fragment analysis (TRF) method or the Flow-FISH method used in experimental settings [24, 25]. However, we participated in an inter-laboratory comparison and achieved coefficients of variation of less than 8%.

Finally, we noted extensive γ-H2A.X staining in the airway bronchial epithelium. Immunostaining of γ-H2A.X in IPF airway epithelial cells has been reported recently by one group that they related to cellular senescence but not to extent of disease [26]. Chromosomal DNA damage in the airway epithelial cells adds to the accumulating evidence [27, 28] that the airways may be important in IPF.

## Conclusions

We have identified changes in telomere length and chromosomal damage in end-stage IPF lungs. Chromosomal damage, measured by γ-H2A.X staining, was associated with parenchymal collapse but not fibrosis suggesting a role for this process in the early stages of IPF progression.
